# Automatic Calculation of Average Power in Electroencephalography Signals for Enhanced Detection of Brain Activity and Behavioral Patterns

**DOI:** 10.3390/bios15050314

**Published:** 2025-05-14

**Authors:** Nuphar Avital, Nataniel Shulkin, Dror Malka

**Affiliations:** 1 Faculty of Education, Bar Ilan University, Ramat-Gan 5290002, Israel; 2 Early Childhood Education, Talpiot College of Education, Holon 5810201, Israel; 3 Department of Natural Sciences, The Open University of Israel, Raanana 4353701, Israel; 4 Faculty of Engineering, Holon Institute of Technology (HIT), Holon 5810201, Israel; drorm@hit.ac.il

**Keywords:** EEG, FIR, IIR Chebyshev filter

## Abstract

Precise analysis of electroencephalogram (EEG) signals is critical for advancing the understanding of neurological conditions and mapping brain activity. However, accurately visualizing brain regions and behavioral patterns from neural signals remains a significant challenge. The present study proposes a novel methodology for the automated calculation of the average power of EEG signals, with a particular focus on the beta frequency band which is known for its pronounced activity during cognitive tasks such as 2D content engagement. An optimization algorithm is employed to determine the most appropriate digital filter type and order for EEG signal processing, thereby enhancing both signal clarity and interpretability. To validate the proposed methodology, an experiment was conducted with 22 students, during which EEG data were recorded while participants engaged in cognitive tasks. The collected data were processed using MATLAB (version R2023a) and the EEGLAB toolbox (version 2022.1) to evaluate various filters, including finite impulse response (FIR) and infinite impulse response (IIR) Butterworth and IIR Chebyshev filters with a 0.5% passband ripple. Results indicate that the IIR Chebyshev filter, configured with a 0.5% passband ripple and a fourth-order design, outperformed the alternatives by effectively reducing average power while preserving signal fidelity. This optimized filtering approach significantly improves the accuracy of neural signal visualizations, thereby facilitating the creation of detailed brain activity maps. By refining the analysis of EEG signals, the proposed method enhances the detection of specific neural behaviors and deepens the understanding of functional brain regions. Moreover, it bolsters the reliability of real-time brain activity monitoring, potentially advancing neurological diagnostics and insights into cognitive processes. These findings suggest that the technique holds considerable promise for future applications in brain–computer interfaces and advanced neurological assessments, offering a valuable tool for both clinical practice and research exploration.

## 1. Introduction

Electroencephalography (EEG) allows for the observation of the brain’s internal electrical activity, where neuronal signals related to cognitive and motor functions produce microvolt-scale potentials. This non-invasive method uses a wearable headset with electrodes placed on the scalp to capture and monitor neural activity in real time. Each electrode functions as an independent channel, capturing oscillatory rhythms that offer critical insights into brain function. EEG amplifiers boost these weak signals for further analysis [1,2] and a signal processing unit digitizes the data for computational evaluation [3]. Due to its non-invasive nature and minimal risk to subjects [4], EEG is extensively used for continuous brain monitoring, especially in clinical neurology.

The primary goal of EEG signal analysis is to help clinicians detect abnormal neural patterns linked to neurological disorders like epilepsy and to locate brain lesions or tumors [5]. In addition to traditional clinical uses, EEG has found applications in various other fields. For example, it assists in diagnosing sleep disorders like insomnia and sleep apnea [6] and is increasingly used in technologies such as driver drowsiness detection systems to reduce fatigue-related accidents [7]. Moreover, EEG-based brain–computer interfaces (BCIs) are being investigated for home automation, allowing users to control Internet of Things (IoTs) devices through neural commands. These advancements promise to enhance independence for individuals with disabilities or age-related mobility issues [8].

The versatility of EEG signals makes it essential not only in clinical environments but also in cognitive neuroscience and human–computer interaction research. Real-time analysis of neural oscillations allows researchers to study cognitive load, decision-making processes, and emotional states. However, challenges remain in signal processing and interpretation because raw EEG data are prone to artifacts such as electromyographic interference, eye movements, and environmental noise. The MATLAB EEGLAB toolbox [9] is a leading software platform for EEG analysis, providing tools for data preprocessing, visualization, and artifact removal. Independent component analysis (ICA), a core algorithm in EEGLAB, effectively separates and removes artifacts caused by muscle activity [10]. Additional plugins improve artifact rejection by using parameters like maximum flat channel duration, high-frequency noise thresholds, and inter-channel correlation metrics [11].

A key challenge in EEG analysis is optimizing digital filters to isolate specific frequency bands, delta (0.5–4 Hz), theta (4–8 Hz), alpha (8–13 Hz), beta (13–30 Hz), and gamma (>30 Hz), each associated with different neurological states. For instance, activity in the beta band is linked to cognitive tasks that demand sustained attention [12]. Poorly chosen filters can distort signals or fail to remove noise, undermining the validity of the analysis. Choosing the right filter involves balancing the advantages and disadvantages of finite impulse response (FIR) and infinite impulse response (IIR) designs. FIR filters provide a linear phase response but require more computational power because they often need a higher filter order [13]. In contrast, IIR filters can achieve similar attenuation with fewer coefficients but may cause phase distortions [14]. Among IIR filters, Butterworth filters offer a maximally flat passband, whereas Chebyshev filters provide a steeper roll-off but introduce a ripple in the passband [15].

## 2. Experimental Materials and Theory

### 2.1. Experiment and Equipment

A total of 22 students volunteered for this study, which involved watching 2D content for 584 s. The 2D content consisted of a recorded lecture from the image processing lab course demonstrating how to remove noise from images using a median filter.

Neural activity was recorded using a BioSemi ActiveTwo wired EEG system, with signal acquisition managed via the SystemPlus Evolution software (version 1.4) platform. Data were collected from a 64-channel EEG headset at a sampling rate of 500 Hz, capturing neural activity across five key brain regions: frontal, parietal, occipital, left temporal, and right temporal.

The primary goal of the study was to identify the most active brain frequency band during engagement with the 2D content. The relevant frequency bands were defined as delta (1–4 Hz), theta (4–8 Hz), alpha (8–13 Hz), beta (13–30 Hz), and gamma (>30 Hz) [16,17]. Previous research comparing brain activity during virtual reality (VR) experiences versus 2D video viewing has indicated that the beta band shows the highest levels of activity [18].

### 2.2. Data Acquisition and Preprocessing

**Figure 1:**
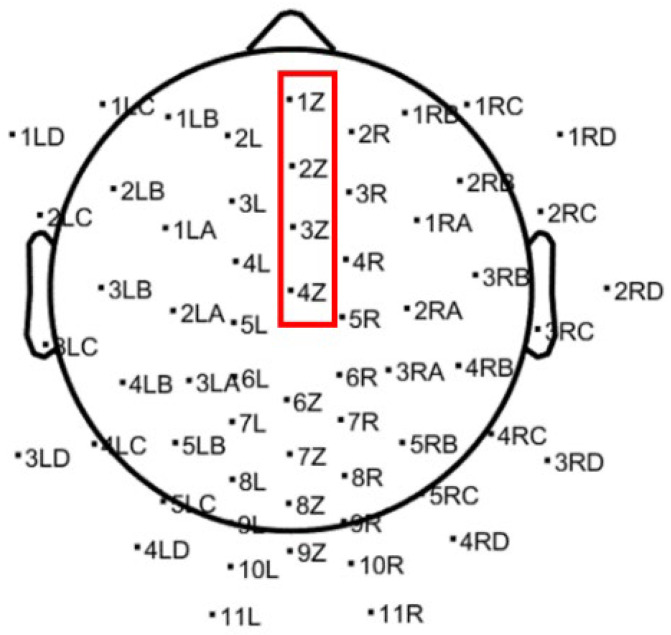
Scalp electrode placement and brain map for EEG recording with 4 marked (red color) channels are used as an example.

The first step of this work involved data loading and preprocessing, following a structured sequence. Initially, the channel locations on the scalp were mapped, as shown in Figure 1, followed by re-referencing the data to minimize noise and artifacts that could arise from referencing all electrodes to a common reference electrode. Subsequently, ICA was applied using the “Infomax” algorithm [19], allowing multivariate signals to be decomposed into statistically independent components. This approach helps in isolating neural activity from non-neural artifacts, such as muscle movements or eye blinks. To further improve signal quality, additional plugins were used to automatically remove channels that remained flat for more than 10 s. The standard deviation of high-frequency noise was capped at 4, and a minimum correlation of 0.7 between neighboring channels was set. A low correlation between adjacent channels typically indicates poor signal quality, often due to poor electrode contact with the scalp, resulting in inaccurate recordings of brain activity. After preprocessing, 3 noisy or faulty channels were removed, leaving 61 out of the original 64 channels available for analysis.

**Figure 2:**
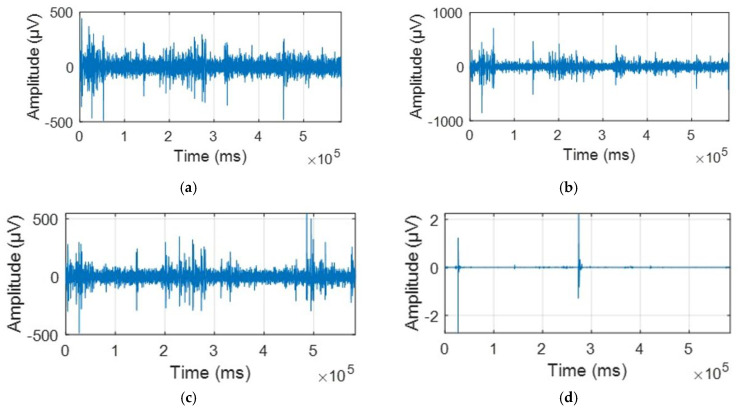
An example of the selected four random channels in the time domain before preprocessing and ICA is presented in the following order: (**a**) 1z. (**b**) 2z. (**c**) 3z. (**d**) 4z.

Time-domain representation plots of EEG signals from 4 random channels (1Z, 2Z, 3Z, and 4Z, shown in Figure 1) in the frontal and parietal regions before and after preprocessing are presented in Figure 2 and the key differences are analyzed later on.

**Figure 3:**
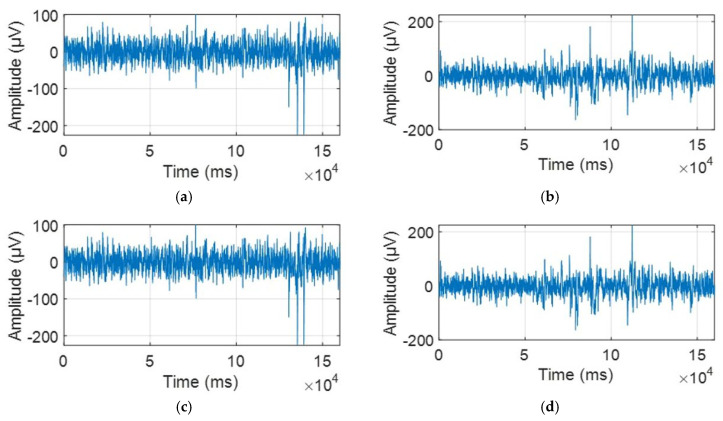
An example of the selected four random channels in the time domain after preprocessing and ICA is presented in the following order: (**a**) 1z. (**b**) 2z. (**c**) 3z. (**d**) 4z.

Time-domain representations of EEG signals from four random channels (1Z, 2Z, 3Z, and 4Z) are displayed in Figure 2 and Figure 3, showing the signals before and after preprocessing, respectively.

There are two key distinctions between the time-domain EEG signal plots before and after preprocessing. Firstly, the duration of the signals is notably different as the original signal lasted 584 s, while the preprocessed signal was reduced to 160 s. This reduction is attributed to the removal of a significant portion of the data which was contaminated by artifacts caused by eye blinks, muscle movements, and other sources of noise.

Secondly, the magnitude of the signals varies significantly. The original data displayed several peaks with considerably higher amplitudes, which were influenced by eye blinks, muscle movements, and extraneous noise, interfering with the accurate representation of neural activity. A striking example of this is channel 4, which exhibited an amplitude of nearly 0.02 V, an implausibly high value for pure neural activity.

**Figure 4:**
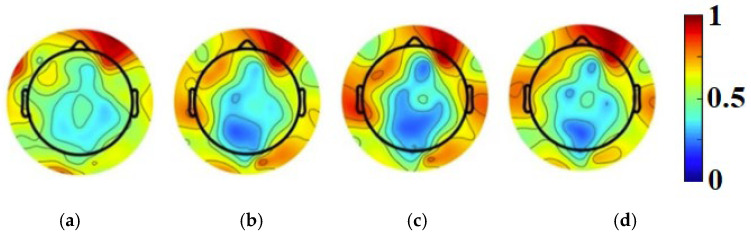
Normalized PSD colormap of brain activity in beta frequency spectrum post filtering: (**a**) 14 Hz; (**b**) 20 Hz; (**c**) 25 Hz; and (**d**) 29 Hz.

Following this analysis, the next step involved identifying the most active brain frequency spectrum of the participant while engaging with the 2D content. Utilizing the ERPLAB plugin for EEG signal filtering, we determined that the beta band (13–30 Hz) was the most active frequency range as it exhibited a significantly higher power spectral density (PSD) compared to other frequency bands. The normalized PSD colormap illustrating brain activity within the beta frequency band is presented in Figure 4.

**Figure 5:**
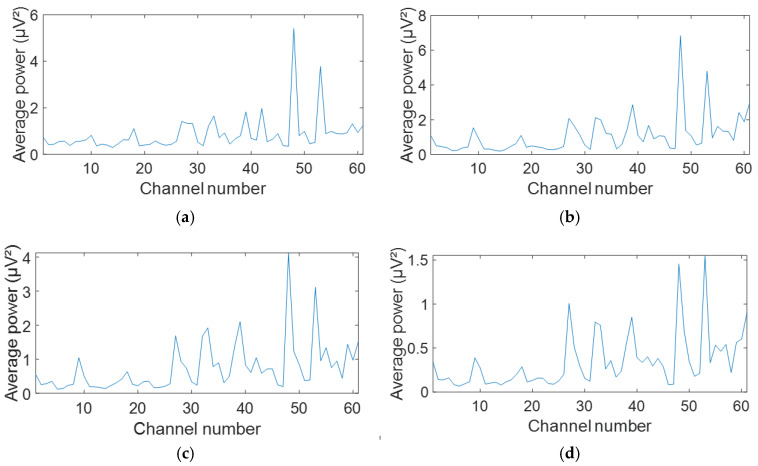
An average power of all 61 channels across beta band frequencies: (**a**) 14 Hz; (**b**) 20 Hz; (**c**) 25 Hz; and (**d**) 29 Hz.

According to Figure 4, the highest neural activity was in the right and the left side of the frontal region. The graphs in Figure 5 show the average power in each channel at the corresponding frequencies from Figure 4.

### 2.3. Digital Filters

**Figure 6:**
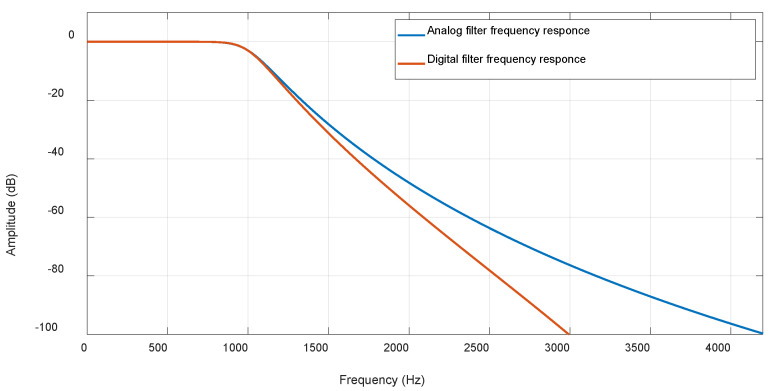
Comparison between analog and digital 8th order Butterworth filters with cutoff frequencies of 1 KHz.

To begin, we should consider the reasons digital filters are typically preferred over their analog counterparts, particularly the significance of sampling signs before the application of digital filtering. In cases where a perfectly flat passband is desired, analog filters can still display a ripple of about 1%. In contrast, digital filters can achieve a remarkably flat response within the passband, often maintaining a ripple of only around 0.02%. This efficiency is rooted in the principles of digital filter theory [20,21,22,23]. Furthermore, while it is true that analog filters tend to attenuate stopband signals more swiftly, the advantages offered by digital filtering frequently make it the superior choice. To illustrate these points effectively, we can refer to Figure 6, which compares an analog filter with a digital 8th-order Butterworth filter, both configured with a cutoff frequency of 1 kHz.

These characteristics were central to our work as artifact rejection and accurate EEG data retrieval were crucial. Digital filters are typically classified in two ways: by impulse response (e.g., FIR or IIR) and by frequency response (e.g., Chebyshev or Butterworth). In this study, we focused on the frequency response classifications for use in our optimization algorithm, specifically the Ebyshev and Butterworth filters.

The Chebyshev filter was applied when a faster roll-off was required, albeit at the cost of passband ripple in the frequency response. There is an inherent trade-off between the passband ripple and roll-off speed because as the ripple increases the roll-off sharpens. The ripple, expressed as a percentage of the passband signal amplitude, is typically set around 0.5%, which is considered a good balance. This value was chosen to strike a practical compromise between maintaining signal fidelity and achieving sharper transition bands. A 0.5% ripple introduces minimal distortion to the EEG signals, preserving critical neural features while effectively suppressing nearby artifacts. A ripple set to 0% produces a maximally flat, or Butterworth, filter. As expected, Butterworth filters offered a smooth passband at the cost of a more moderate roll-off speed.

**Figure 7:**
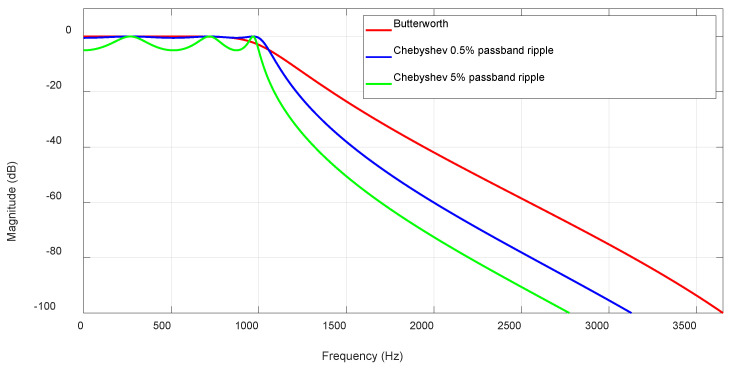
Magnitude response of 6th order 1 KHz cutoff frequency Butterworth, Chebyshev with 0.5% passband ripple, and Chebyshev with 5% passband ripple digital filters.

Figure 7 presents a comparison between these filter types, illustrating the trade-off between the roll-off speed and passband ripple. It shows the magnitude responses of three 6th-order digital filters with a 1 kHz cutoff frequency: a Butterworth filter, a Chebyshev filter with 0.5% ripple, and a Chebyshev filter with 5% ripple.

The second type of digital filter classification is by their impulse response, which is represented as h[k]. There are two types: finite impulse response (FIR) and infinite impulse response (IIR).

The relationship between the input and output signals of an FIR filter is defined by the following convolution sum:

(1) $$y\left[n\right]=\sum_{k=0}^{N-1}h\left[k\right]x[n-k]$$

where y[n] is the output of the filtered signal and x[n] is the input signal, and N refers to the number the filter coefficients.

The main advantage of the implementation of this filter lies in its stability. No matter how high the number of coefficients, it will always be stable. In addition to that, the phase response of this filter is always linear. However, these filters have some flaws such as a moderate cutoff speed and a significantly higher coefficient number when compared to IIR filters.

The input and output signals to the IIR filter are defined by the following convolution sum:

(2) $$y\left[n\right]=\sum_{k=0}^{\infty }h\left[k\right]x[n-k]$$

In practice, it is not possible to calculate the output of the IIR filter because N is infinite. So alternatively, the practical IIR filtering equation uses feedback of previous output values. Thus, the relationship between the input and output signals of an IIR filter is described by the following recursive difference equation, incorporating both current and past inputs as well as past outputs:

(3) $$y\left[n\right]=\sum_{k=0}^{N}a_{k}x\left[n-k\right]-\sum_{k=1}^{M}b_{k}y[n-k]$$

where b_k_ is called the feedback coefficient and involves the past output values and determines their contribution to the current output.

IIR filters have several positive traits such as a sharp cutoff, fewer coefficients than FIR filters, and higher computation speed. But it all comes at the cost of the non-linear phase response and a risk of instability.

In this case, the primary concern when using IIR filters is the potential for instability. However, this is effectively mitigated by employing a zero-phase filtering technique [24], which eliminates phase distortion and ensures signal integrity by processing the data in both forward and reverse directions. This approach is implemented using the fulfill*t()* function in MATLAB, which applies the filter twice to maintain the signal’s temporal alignment.

### 2.4. Optimization Algorithm

The algorithm begins with the start phase, followed by loading EEG data using EEGLAB, where the EEG dataset is imported into the EEGLAB environment for subsequent analysis. In the data preprocessing step, initial preprocessing tasks are performed, including channel selection, re-referencing, and down-sampling if necessary. This ensures that the data are appropriately configured for further processing. Next, during the noise and artifact detection phase, common artifacts, such as eye movements and muscle noise, are identified and removed using techniques like ICA. This step is crucial for enhancing the quality of the EEG data.

Once the data are cleaned, the algorithm proceeds to determine the optimal filter type and order. Here, the signal is analyzed to identify the most suitable filter types, specifically FIR, IIR Butterworth, and IIR Chebyshev, along with their respective orders. For this analysis, a focus is placed on minimizing average power within the specified brain frequency band (beta: 13–30 Hz) in a specific channel with high neural activity (channel 1RB was chosen for the example). It is important to emphasize that this method is not limited to a single channel; rather, it is fully applicable to all EEG channels and is used to generate the full-brain PSD colormap reflecting activity across different regions.

The FIR filter is set with an order range of 200–300, the IIR Butterworth filter with an order range of 4–9, and the IIR Chebyshev filter with a 0.5% passband ripple and an order range of 2–9. The rationale behind these specific filter orders will be discussed later in the optimization algorithm results and analysis section. Following the optimization, the filter design and application step is executed, wherein the selected optimal digital filter is applied to the EEG data.

**Figure 8:**
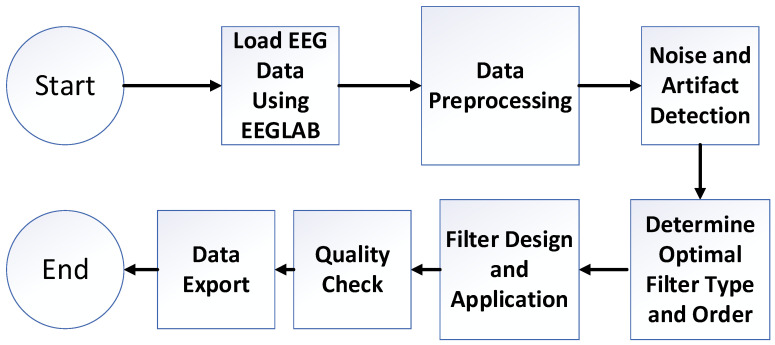
Block diagram of EEGLAB preprocessing and optimization algorithm.

The algorithm then moves to the quality check phase, where the quality of the filtered data is verified. This ensures that artifacts have been minimized and the integrity of the signal is preserved. Finally, the filtered EEG data are saved in the data export step, making it ready for further analysis. The entire process is summarized in Figure 8, illustrating the flow of the algorithm including all its steps.

## 3. Comprehensive Analysis of Optimization Algorithm Results

**Figure 9:**
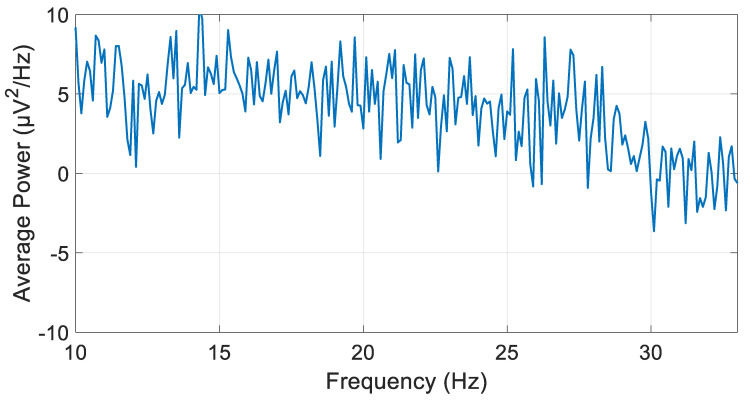
Welch’s PSD calculation method graph of preprocessed and unfiltered signals across beta band frequencies.

Before iterating through the various filter types and orders, the average power of the preprocessed, unfiltered signal in the beta frequency band (channel 1RB) was calculated to facilitate a subsequent comparison with the signal processed using the optimal filter type and order. The average power was found to be 133.03 µV^2^. Figure 9 illustrates the PSD calculation of the preprocessed and unfiltered signal across beta band frequencies, employing Welch’s method.

The average power value of the filtered signal is desired to be lower than the unfiltered signal since the filtered signal contains only the power from the specified band, which is usually less than the total power of the unfiltered signal that includes contributions from adjacent frequencies and noises [25]. It is important to emphasize that each digital filter was designed in a way that it will be maximally flat in the beta band as accurately as possible so that the low value of the average power is not caused by the EEG data attenuation in that band.

### 3.1. IIR Butterworth Filter

**Figure 10:**
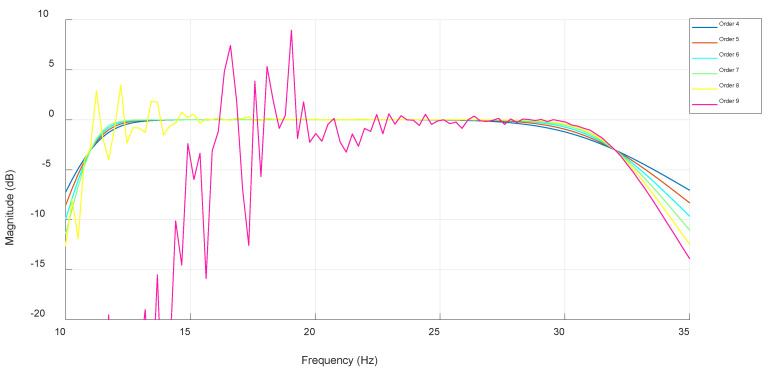
IIR Butterworth filter magnitude response from order 4 to 9.

Experimenting showed that the optimal lower cutoff frequency of this filter was set to 11 Hz while the higher cutoff frequency of this filter was set to 32 Hz since it has a moderate roll-off. Therefore, if the cutoff frequencies are set closer to the accurate beta frequency band, the signals belonging to the edge frequencies will be attenuated. The lowest filter order was set to for, the reason being that lower orders have too much of a mild roll-off, while the highest filter order was set to nine since higher orders will surely be unstable. The magnitude response of each filter order is shown in Figure 10.

**Figure 11:**
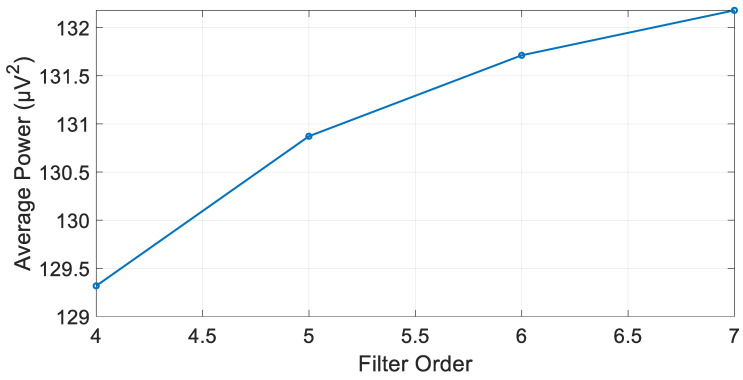
Filtered signal average power at each stable IIR Butterworth filter order.

As can be recognized, the max filter order at which the filter is still stable is seven. The algorithm can recognize unstable filter orders and discard them while calculating the average power of the filtered signal. Figure 11 presents the filtered signal average power at each stable filter order. The optimal filter order that yielded a filtered signal with an average power of 129.319 µV^2^ was determined to be four, as can be seen in Figure 11.

### 3.2. IIR Chebyshev Filter with 0.5% Passband Ripple

**Figure 12:**
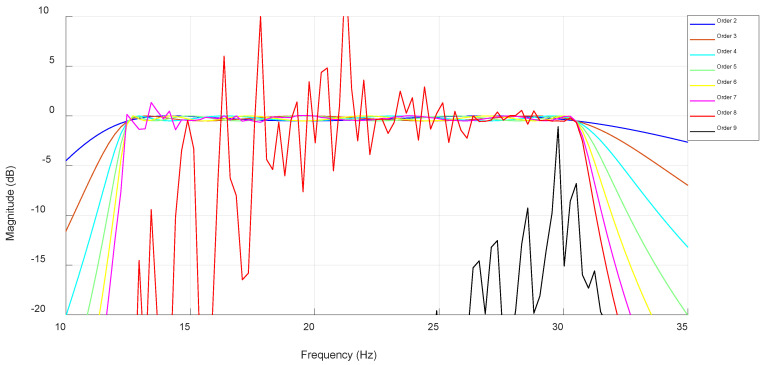
IIR Chebyshev filter magnitude response from order 2 to 9.

The lower cutoff frequency of this filter was set to 12.5 Hz while the higher cutoff frequency of this filter was set to 30.5 Hz. These frequencies are very close to the beta band frequency range because the combination of IIR and Chebyshev produces a very steep roll-off. The lowest filter order was set to two because this order has a fast enough roll-off, while the highest filter order was set to nine since higher orders will be surely unstable. The magnitude response of each filter order is shown in Figure 12.

**Figure 13:**
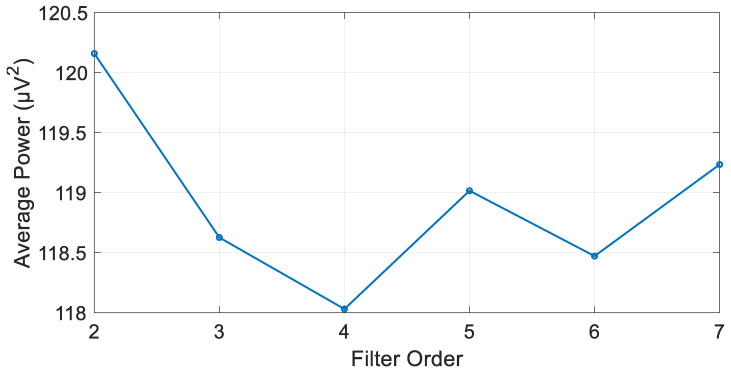
Filtered signal average power at each stable IIR Chebyshev filter order.

The highest filter order at which the filter is still stable is set by the algorithm to seven. When the filter was applied to the signal it wielded an average power of 118.03 µV^2^. Figure 13 shows the average power of the signal when passed through each stable filter order.

### 3.3. FIR Filter

**Figure 14:**
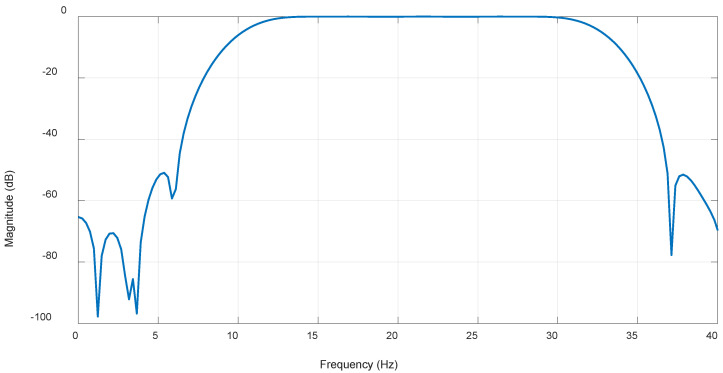
FIR filter with optimal order of 213 magnitude response.

**Figure 15:**
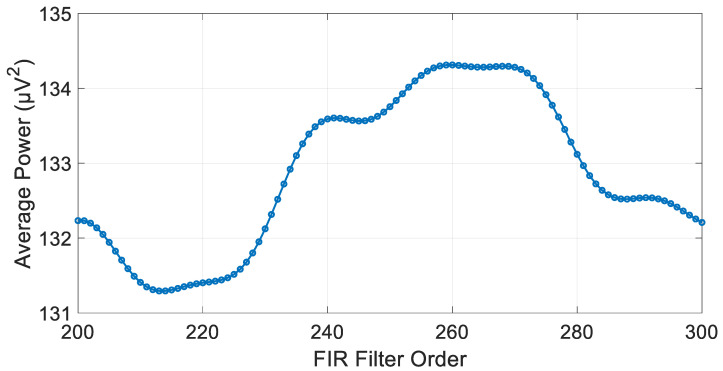
Filtered signal average power at each FIR filter order.

It is well-known that this type of filter is significantly less efficient in terms of computational power and exhibits a relatively slow roll-off compared to IIR filters. However, it is always stable, allowing for the selection of very high filter orders to achieve a flatter response in the passband. Through experimentation, it was determined that the optimal filter order range is between 200 and 300 as the lower limit provides a relatively flat passband. The filter cutoff frequencies were set to 10 Hz and 33 Hz, with an optimal filter order established at 213, resulting in a signal power of 131.295 µV^2^. Figure 14 and Figure 15 illustrate the magnitude response of the optimal filter order and the average power of the filtered signal at each filter order, respectively.

**Figure 16:**
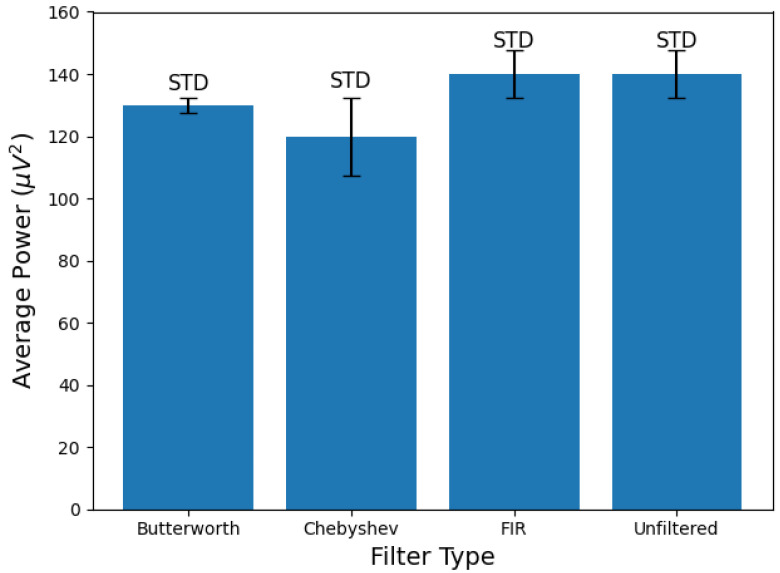
Comparison of the average power of EEG signals in channel 1RB across the beta frequency band after applying different digital filters (Butterworth, Chebyshev, FIR) and when it is unfiltered. Error bars represent the STD of the average power.

After the algorithm iterated through all the different filter types and orders, it automatically selected the optimal digital filter based on the lowest average power of the filtered EEG data in channel 1RB. This outcome is expected due to the fast roll-off characteristic of this filter type, which facilitates effective noise and artifact attenuation. However, it is important to note that this advantage comes with the trade-off of passband ripples which can distort the originally acquired EEG data. This factor must be considered when accurate data reception is critical. Figure 16 presents a bar plot summarizing the average power values of the unfiltered original EEG data alongside the same data filtered by each filter type at their optimal orders across the beta frequency band in channel 1RB. In this figure, the standard deviation (STD) range of the average power for each filtered and unfiltered signal is indicated by a horizontal line with vertical bars at both ends.

**Figure 17:**
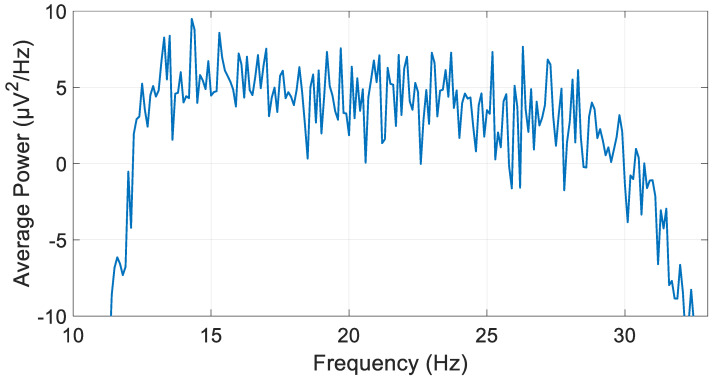
Welch’s PSD calculation method graph of filtered signal across beta band frequencies.

After the implementation of the algorithm optimal filter type and order (fourth order IIR Chebyshev) on the EEG data, a comparison between the original EEG data and the filtered data was made to ensure that the data were filtered well and that the average power of the filtered signal was smaller than the original data uniformly across all of the beta band. Figure 17 shows the filtered signal PSD across the beta frequency band.

**Figure 18:**
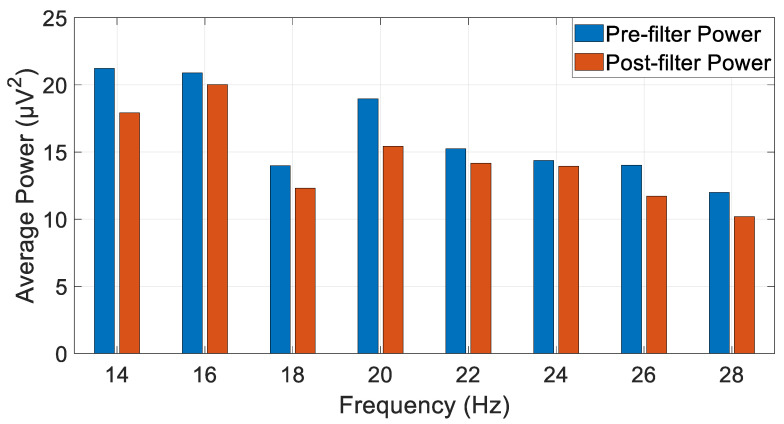
The average power of the filtered and unfiltered signal in chosen frequencies from the beta frequency band.

When the graph is compared to the PSD of the unfiltered signal, first and foremost it can be seen that the shape of the filtered data corresponds to the beta band frequencies. Additionally, the PSD of the filtered signal depicts smaller PSD values across the relevant frequencies which points to the filtration of noises and interference from other undesired frequencies. An additional representation of the average power of unfiltered and filtered signals in chosen frequencies inside the beta band is shown in Figure 18.

The graph shows the lower average power of the filtered signal when compared to the unfiltered signal uniformly across all of the chosen beta band frequencies.

### 3.4. Algorithm Optimization and Comparative Performance

Our automated approach substantially accelerates the analysis process relative to the conventional manual methods employed in EEGLAB. To substantiate the advantages of our method over the traditional EEGLAB approach, we performed a comparative analysis of processing times as a function of subject sample size (N). Although both methodologies follow analogous preprocessing procedures, their execution times differ markedly, as detailed in Table 1.

**Figure 19:**
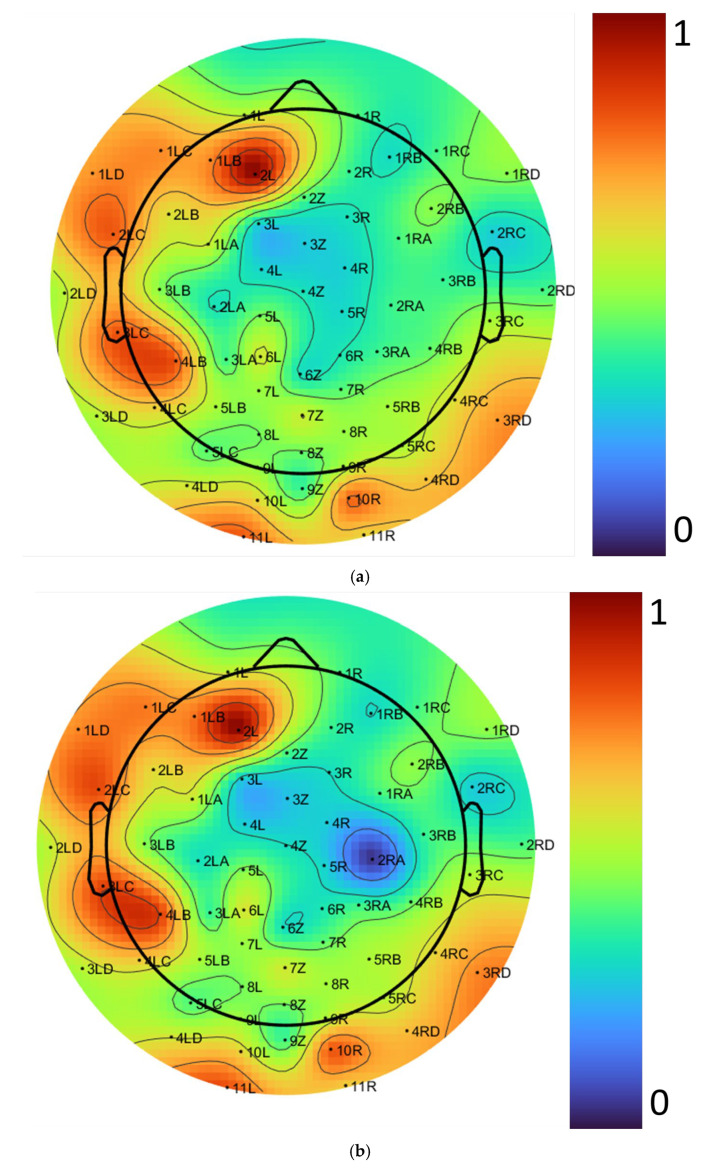
Topographical distribution of EEG electrode placement for a 64-channel EEG cap layout with normalized power distribution at 25 Hz: (**a**) EEGLAB tool, and (**b**) automatic algorithm.

To evaluate our algorithm, we analyzed normalized power distributions across electrodes at 25 Hz, a frequency within the beta band that is crucial for cognitive engagement [27,28]. The results using the EEGLAB tool are presented in Figure 19a, while the results from our automated algorithm are shown in Figure 19b. In these figures, the color bar represents normalized power values ranging from 0 to 1.

**Figure 20:**
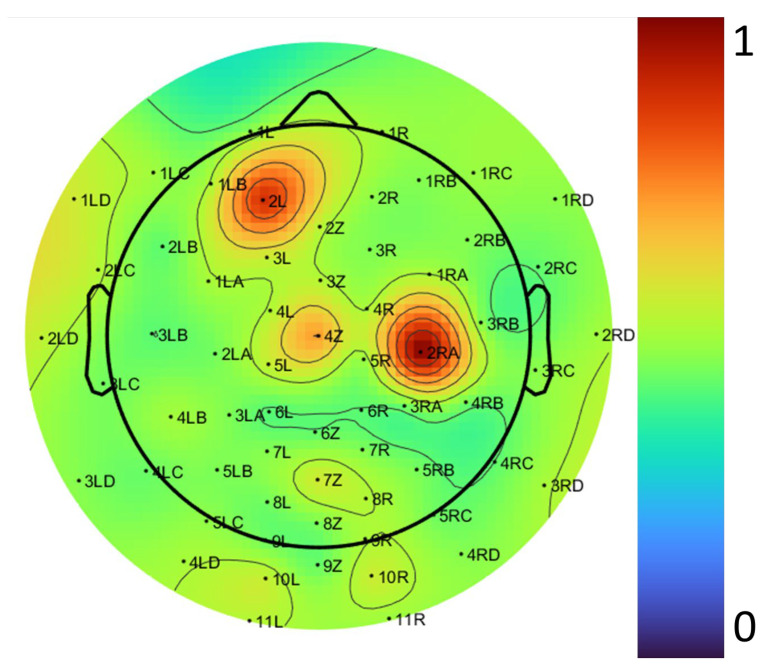
The difference in the topographical distribution of EEG electrode placement between the EEGLAB tool and the automated algorithm at 25 Hz.

To compare the performance of the proposed algorithm with the EEGLAB tool, we conducted an evaluation using a test case at 25 Hz, which resulted in a reduction in the average power difference between the two methods. At this frequency, significant differences in average power were observed, as shown in Figure 20. The improvements were particularly evident in the following two specific regions: channel 2RA, located over the left frontal region, a region involved in language processing, attention mechanisms, and other cognitive functions, and channel 2L, located over the frontal lobe, which is crucial for decision-making, problem-solving, and planning. These improvements highlight the importance of these regions for further research in cognitive neuroscience and related fields. This proves that our proposed algorithm yields better results by not only mapping brain activity more accurately in terms of location but also by capturing activity levels with greater precision. This is crucial for research in brain function analysis. Our method demonstrates superiority by being faster and more accurate than the filtering process in the EEGLAB tool, which is less precise and more time-consuming.

Table 2, which shows a comparison between advanced machine learning (ML) methods and our approach, highlights the distinct advantages of our method. Unlike convolutional neural networker (CNN) transformer-based models (such as a dual-channel hybrid convolutional transformer generative adversarial network (DHCT-GAN) [29] or a convolutional transformer network (CTNet) [30]) that require GPU acceleration for speed, our method runs efficiently on standard CPUs, making it cost-effective and accessible for labs or institutions without high-end hardware. Deep learning models consume substantial memory and power, especially during training and inference. In contrast, our approach minimizes memory usage and energy consumption, making it ideal for embedded systems, portable EEG devices, and real-time clinical settings. While ML methods can achieve fast classification times with a GPU, our algorithm delivers comparable speed without relying on hardware acceleration and maintains sub-30 s classification times even as data volume increases. Furthermore, our approach does not rely on complex model training or dataset-specific optimization, making it more generalizable and easier to deploy across different EEG platforms and experiments.

## 4. Conclusions

In this study, we developed an optimization algorithm to identify the optimal digital filter for calculating the average power of EEG signals within a specific brain frequency band, particularly in a highly active channel. By utilizing the MATLAB EEGLAB toolbox and incorporating various filtering techniques such as FIR, IIR Butterworth, and IIR Chebyshev, we enhanced the precision of EEG signal analysis by effectively removing noise and artifacts. Specifically, in our analysis of EEG data sampled from subjects engaged with 2D content, our findings revealed that the IIR Chebyshev filter with a 0.5% passband ripple and an order of four delivered the best performance. This filter achieved a lower average power in the beta band compared to both the original EEG data and other filter types and orders. The implications of our results extend beyond signal processing alone; they underscore the critical importance of selecting appropriate digital filters for EEG signal analysis, which can significantly affect the accuracy and reliability of findings. Accurate EEG signal analysis is essential for understanding behavioral patterns, particularly in educational contexts. By enhancing EEG signal clarity, we can improve the visualization and interpretation of cognitive engagement, attention levels, and instances of mind wandering among students during learning tasks. This method enhances our ability to map brain activity accurately, providing insights into functional brain regions associated with various cognitive processes. For instance, our optimized filter can reveal how attention and cognitive load fluctuate as students interact with educational content, enabling educators to tailor instructional methods to accommodate diverse learning styles and improve outcomes. Moreover, the optimized algorithm can support the development of advanced brain–computer interfaces, which have the potential to revolutionize real-time monitoring and enhance cognitive engagement. By integrating machine learning techniques with our optimization algorithm, we can further refine filter selection, ensuring that each channel’s unique characteristics are accounted for. This advancement could lead to more precise interpretations of neural signals, improving the detection of specific neural behaviors tied to learning and decision-making.

To validate the algorithm’s effectiveness, we compared it to EEGLAB. The results demonstrated that our algorithm operates with significantly shorter computation times and selects filters that yield more accurate EEG analyses, making it particularly valuable for professionals requiring rapid and precise data analysis.

Despite the promising results, this study has several limitations that warrant further investigation. The relatively small sample size may affect the generalizability of the findings to broader or clinical populations. Additionally, the methodology was focused solely on the beta frequency band; future work should extend the analysis to other bands such as alpha and gamma to capture a wider range of neural activity. Finally, more diverse cognitive tasks and real-world scenarios should be considered to validate the robustness of the proposed approach in practical applications.

Future research could explore applying this algorithm across multiple highly active channels to comprehensively analyze interactions between brain regions during various cognitive tasks. Such work could deepen our understanding of functional brain activity and enhance the reliability of neurological diagnostics. Ultimately, this innovation provides a powerful tool for clinical applications and educational research, fostering a deeper understanding of cognitive processes and their behavioral manifestations with the potential to improve educational strategies and interventions.

## Figures and Tables

**Table 1:** Comparison of processing times between EEGLAB tool and automatic–automatic algorithm data analysis across different numbers of subjects (N) [26].

Test Approach	N = 1		N = 10		N = 20	
	Preprocessing	Classification	Preprocessing	Classification	Preprocessing	Classification
EEGLAB	18 min	6 min	~180 min	~60 min	~360 min	~120 min
Automatic algorithm	5 min	<30 s	~50 min	<30 s	100 min	<30 s

**Table 2:** Comparison of advanced ML methods and the proposed automatic algorithm.

Feature	DHCT-GAN [29] and CTNet [30]	Automatic-Algorithm
Hardware Requirements	High (typically requires GPU acceleration)	Low (runs efficiently on standard CPUs)
Computational Cost	High	Low
Memory Consumption	High	Low
Energy Consumption	High	Low
Speed	Fast (with GPU)	Comparable to deep learning (without GPU) and maintains scalability
Generalizability	Can be limited by dataset-specific training	High
Deployment Complexity	High (complex model training and optimization)	Low (simple and straightforward)
Suitability	Applications with access to high-performance computing	Resource-constrained environments, real-time applications, and portable devices

## Data Availability

The data collected and analyzed in this study are not publicly available due to ethical restrictions and privacy considerations. All relevant analyses derived from the data are presented in the manuscript. The raw image data and participant information are securely stored in accordance with institutional guidelines at the Holon Institute of Technology and are not shared to protect participant confidentiality.
